# Effectiveness and influencing factors of online teaching among primary and secondary school teachers in China during the COVID-19: in depth interviews

**DOI:** 10.3389/fpsyg.2023.1256529

**Published:** 2023-09-21

**Authors:** Xiaofang Ma

**Affiliations:** Fuzhou University of International Studies and Trade, Fuzhou, China

**Keywords:** COVID-19, in-depth interviews, open coding, axial coding, selective coding, primary and secondary school teachers, online teaching effectiveness

## Abstract

During COVID-19, online teaching was adopted in many countries and online teaching effectiveness attracted widespread attention. This study used in-depth interviews to explore the factors affecting online teaching efficacy of primary and secondary school teachers from teachers’ perspectives, and used open coding, axial coding, and selective coding to analyze and organize the interview materials. Five thematic elements were finally derived, namely acceptance, professionalism, interactivity, instructional leadership and support, and home-school collaboration. From the teacher’s perspective, acceptance, professionalism, and interactivity are closely related to the individual teacher, while instructional leadership and support, and home-school collaboration are two important external elements. This study explored the influencing factors of teaching effectiveness in different contexts, enriched the theory of teaching effectiveness, and provided practical support for the future development of online teaching.

## 1. Introduction

Teaching effectiveness is an important factor in students’ learning. In the past 3 years, due to receiving the influence of COVID-19, the traditional way of primary and secondary school classes have been broken, presenting a stage of implementation of online teaching, online teaching needs to connect teachers and students through the network, but how effective online teaching is concerned by many educational researchers. How effective was the implementation of online teaching during COVID-19? What factors affect the effectiveness of online teaching? This is not only an important practical issue, but will also be related to the application of information technology in education.

The effectiveness of online teaching refers to the extent to which teachers and students realize their teaching goals through online teaching with the help of internet tools. Regarding the factors affecting online teaching and learning, Garrison et al. (2010) proposed the “Community of Inquiry Framework,” which argues that cognitive presence, teaching presence and social presence are key to judging the effectiveness of teaching and learning, and that these three factors interact with each other. Garrison et al. (2010) explored the relationship between the three elements of the Community of Inquiry Framework: cognitive presence, teaching presence and social presence, and identified teaching presence as a core element of the framework. Shea and Bidjerano (2010) developed the Community of Inquiry Framework, argued that there is a positive relationship between the elements of the original Community of Inquiry Framework model and “learning presence” and constructed a new model that includes cognitive presence, teaching presence, social presence, and learning presence.

Prior research on online teaching efficacy and the factors that influence it has focused on the relationships between elements. In terms of research methodology, there have also been some quantitative studies of influences on the effectiveness of online teaching and learning influences, such as the use of structural equation modeling and other methods to explore the relationship between the various elements, but the depth of quantitative studies focusing on issues may not be sufficient. Additionally, during the period of COVID-19, the discussion about the effectiveness of online teaching will have its unique significance in the special period, therefore, this study used in-depth interviews to explore the perceptions of elementary and secondary school teachers about online teaching during COVID-19, and tried to analyze the factors influencing their online teaching.

Based on the above exploration, the research questions are as below:

- What is the online teaching effectiveness of elementary and secondary teachers during COVID-19?
- What are the factors that influence the effectiveness of online teaching in elementary and secondary teachers during COVID-19?

## 2. Literature review

### 2.1. Definition of online teaching effectiveness

Teacher teaching effectiveness is a composite, multifaceted concept that is often expressed in academic research as “teaching efficacy” or “teaching effectiveness.” Research on teaching efficacy has a long history and is mainly derived from the comprehensive performance of teaching at multiple levels. Due to differences in scholars’ backgrounds and perspectives, there are different understandings, definitions, and interpretations of teaching efficacy. Borich (1988) suggests that effective teaching is judged on the basis of six main aspects: clarity; variety; task orientation; engagement in the learning process; successful classroom management; and teacher-student interactions. If “teaching” is limited to the classroom level, then teaching effectiveness refers to a teacher’s ability to meet or exceed the expected instructional goals by creating a cooperative and orderly classroom atmosphere and a positive and interactive process that meets the actual developmental needs of the students and maximizes their motivation to learn in a particular school context with the students as the center of the instructional activities (Davis, 1989). However, with the development of information technology, teaching has broken through the spatial limitations of classroom teaching, and there were scholars who had put forward their own views on the definition of the effectiveness of online teaching. Noesgaard and Ørngreen (2015) identified 19 different definitions and perspectives on the effectiveness of online learning. Pradana and Amir (2016) defined the effectiveness of online teaching and learning through electronic media as students’ comprehension and assimilation of the lecture material, students’ perception of the instructor’s performance, and students’ satisfaction with the lecture material.

Summarizing the above research, the effectiveness of online teaching is based on the concept of teaching effectiveness, combined with the development of educational technology in the information age, and refers to the extent to which teachers and students are able to achieve their teaching goals through online teaching.

### 2.2. Factors affecting the effectiveness of online teaching

The concept of online teaching effectiveness is multidimensional. Garrison et al. (2010) proposed a community of inquiry framework, which posited that the factors influencing the effectiveness of online instruction encompass cognitive presence, pedagogical presence, and social presence. Garrison et al. (2010) further used the Community of Inquiry framework to explore the causal relationship between cognitive, teaching and social presence, the central role played by teaching presence was confirmed. More than that, Shea and Bidjerano (2010) developed the Community of Inquiry framework, concluded that a positive relationship exists between elements of the Community of Inquiry framework and “learning presence,” formed a new model encompassing teaching presence, social presence, cognitive presence, and learning presence, and explored the relationship between several factors. Synthesizing these studies, the community of inquiry framework provided a reference base for blended and online teaching and learning.

In addition, researchers have explored the factors affecting the effectiveness of online teaching and learning from different perspectives. Jui-Che et al. (2012) raised factors that are thought to affect online learning include: Technology acceptance model, learning psychological process, simulated situation learning and learning theories. Noesgaard and Ørngreen (2015) coded over 110 published papers on the effectiveness of online learning and found that there were three categories of factors that influence the effectiveness of online learning: nature of online learning solution and process, individual factors, and contextual factors, and the key contextual factors are resource (time, technologies: tools and internet), and support (from managers, IT personnel or peers). Abdalla Mohammed and Muhammed Pandhiani (2017) used factor analysis to validate that five major factors can affect the effectiveness of teaching in universities: instructor’s personality, knowledge, teaching ability, marking and grading policy, and course attributes and learning outcomes. Fernandez-Garcia et al. (2019) found that there was a significant effect of teachers’ teaching experience on teaching effectiveness manifested in six teaching skill domains in secondary school. Boonsem and Chaoensupmanee (2020) supposed the factors of physical education teaching effectiveness by comparing students’ opinions on the teaching program at University of Technology, including the sum of six dimensions: (1) the purposes of Physical Education learning; (2) content and Physical Education programs; (3) teaching methods and teaching activities; (4) the personality aspects of Physical Education instructors; (5) equipment and facilities; and (6) assessment and evaluation. Husin et al. (2022) constructed a conceptual framework for influencing teachers’ online teaching effectiveness that encompassed technology factor, learner factor, administrative factor and personal factor. From the students’ perspectives, the factors affecting teachers’ online instruction during COVID-19 were explored, mainly containing students’ interaction, access to quality internet and students online learning competency (Inusah and Debrah, 2021).

By comparing and synthesizing the above studies, online teaching effectiveness can be placed in the developing Community of Inquiry framework, that is, it contains teaching presence, cognitive presence, social presence and learning presence, but there are some overlapping elements and interactions between them, that is to say, there are factors that cannot be exactly classified into each kind of presence. Therefore, the researcher combines the above literature and summarizes the factors affecting teachers’ online teaching into four main aspects: teacher factors, learner factors, technology factors, and administrator factors. Among them, teacher factors mainly include teachers’ personal time management, professional competence, technical level, etc.; learner factors such as learners’ age, independent learning capability, etc.; technological factors such as online teaching platforms and equipment; and managerial factors such as school support.

Taken together, most of the previous studies used quantitative research methods to explore the influencing factors of online teaching efficacy or to analyze online teaching efficacy from students’ perspectives. This study used in-depth interviews to examine teachers’ online teaching efficacy and its influencing factors during COVID-19 from teachers’ perspectives, and was able to explore more deeply what online teaching means to teachers.

## 3. Research design

### 3.1. Instrument

The design of this study was based on semi-structured in-depth interviews and thematic analysis in order to explore the factors that influence the effectiveness of online teaching and learning for primary and secondary school teachers. The study aimed to demonstrate the key influences and implementation effectiveness of online teaching from the teachers’ perspective. To achieve this goal, the researcher developed a semi-structured interview outline. The main interview questions focused on the following areas:

1. Teachers’ perspectives on the implementation of online teaching during COVID-19.
2. What are the pedagogical methods used by teachers in online teaching?
3. How effective was the implementation of online teaching?
4. How the teachers guided the students to learn independently?
5. What difficulties do teachers have in teaching online?
6. What aspects of online teaching can be improved?

In addition, the interviewer asked follow-up questions based on the respondents’ answers.

### 3.2. Participants

Quick and Hall (2015) suggested that sample sizes for qualitative research are usually between 4 and 50 due to the amount of data collected. Corbin and Strauss (2007) also suggested that 5 or 6 h of interviews can provide enough data to saturate the study. In addition, participants should be fully utilized so that they are best represented and informed about the research topic. In terms of data, the data should be adequate and enable a rich description of the phenomenon. On this basis, 12 primary and secondary school teachers participated in the interviews and thus it can be said that the sample size of this study adequately meets the recommended requirements.

Twelve interviewees were selected for this study for the following reasons: first, the interviewees were from different regions, interviewees came from eight different provinces, each with a different level of educational and economic development, in order to understand the different aspects of online teaching and learning in different regions. Second, the interviewees covered primary and secondary school teachers of different genders, teaching ages, subjects, and schools, covering as many different experiences as possible, trying to ensure that the research subjects were representative and able to collect as comprehensive an array of online teaching experiences as possible, and then exploring the effectiveness of online teaching. And finally, the respondents were from rural and urban areas, half of the respondents were teachers from urban areas and the other half were from rural areas in order to understand the different contexts of online teaching in urban and rural areas. Specific information about the interviewees is provided in Table 1.

**Table 1:** Information of participating teachers.

Teacher	Gender	Teaching experience	Teaching stage and subjects	Province	Urban/Rural
Teacher 1	Female	15 years	Primary English	Shandong	Rural
Teacher 2	Male	3 years	Secondary Physics	Fujian	Rural
Teacher 3	Female	6 years	Secondary Chinese	Fujian	Urban
Teacher 4	Female	2 years	Primary Chinese	Fujian	Urban
Teacher 5	Male	5 years	Secondary Chinese	Jiangxi	Rural
Teacher 6	Male	7 years	Primary Chinese	Zhejiang	Urban
Teacher 7	Female	3 years	Secondary History	Hebei	Rural
Teacher 8	Female	10 years	Secondary History	Jiangsu	Rural
Teacher 9	Female	8 years	Primary Mathematics	Hubei	Urban
Teacher 10	Male	16 years	Primary English	Anhui	Urban
Teacher 11	Female	2 years	Primary Morality and Rule of Law	Shandong	Urban
Teacher 12	Female	6 years	Secondary General Studies Program	Hebei	Rural

In order to have a clearer picture of the distribution of the interviewees in terms of gender, teaching experience, teaching stage, and disciplines, the demographic characteristics of the interviewees are now presented as shown in Table 2.

**Table 2:** The demographic characteristics of participating teachers.

Characteristics	Attributes	Distributions	Proportions
Gender	Male	5	41.67%
	Female	7	58.33%
Teaching experience	1–5 years	5	41.67%
	6–10 years	5	41.67%
	More than 11 years	2	16.67%
Teaching stage	Primary	6	50.00%
	Secondary	6	50.00%
Subjects	Chinese	4	33.33%
	Mathematics	1	8.33%
	English	2	16.67%
	Physics	1	8.33%
	History	2	16.67%
	Morality and Rule of Law	1	8.33%
	General Studies Program	1	8.33%

As shown in Table 2, in terms of gender distribution of teachers, there are 5 male teachers and 7 female teachers, which is relatively balanced. From the interviewees’ student grade level, there were 6 elementary school teachers, 6 secondary school teachers, covering the subjects of Chinese, Mathematics, English, Physics, History, Ethics and Rule of Law, and General Studies Program. In terms of teaching experience of the interviewees, there were 5 teachers with 1–5 years of teaching experience, 5 teachers with 6–10 years of teaching experience, and 2 teachers with more than 11 years of teaching experience.

### 3.3. Data collection

In this study, we conducted in-depth interviews with 12 elementary and secondary school teachers on their online teaching effectiveness and factors. Interviews were conducted with 12 teachers between April 2020 and August 2023 one after another. Each interview lasted between 30 and 60 min and was audio-recorded with the consent of the interviewees and a transcript of the interview was produced. In order to protect the privacy of the interviewees, they were anonymously coded, i.e., numbered in the order in which they were interviewed and categorized as teachers from 1 to 12.

### 3.4. Data analysis

The researcher used grounded theory as a guide to analyze the interview data. The theory provides a systematic process for identifying categories and their associations, and the coding process was inductive and involved constructing a theory by analyzing the data (Urquhart, 2012). We used open coding, axial coding, and selective coding as recommended by Creswell (2009) and Strauss and Corbin (1998). The first step was to collect data based on a list of questions. As the researcher reviewed the data, recurring concepts became apparent and were grouped into “categories.”

As the researcher reviewed the data, recurring concepts became apparent and were categorized. The researcher coded each interviewees’ language sentence-by-sentence to label concepts related to factors influencing the effectiveness of online teaching and learning. And then, similar labels were conceptualized, and the concepts were summarized to categories were refined. After open coding, the researcher summarized the five categories of “acceptance,” “professionalism,” “interactivity,” “instructional leadership and support” and “home-school collaboration.” And the coding number and percentage of codes shown in Table 3.

**Table 3:** Example of coding process.

Open coding	Axial coding	Selective coding	Number of codes	Proportions
*“Some software features are quite good, such as Ding Talk, which is quite powerful in terms of checking in, monitoring students’ listening and grading assignments, etc.”*	Perceived usefulness	Acceptance	10	2.36%
*“Some veteran teachers may be intimidated by learning to use the software.”*	Perceived ease of use			
*“As a teacher and a parent, I think it was a good way to teach online.”*	Attitudes toward online teaching			
*“Teachers are more attentive and detailed when teaching online than offline.”*	Professional attitude	Professionalism	147	34.75%
*“Considering many factors, we changed the teaching content and chose to explain some of the knowledge points in the general knowledge of Chinese ancient culture.”*	Professional capacity			
*“After experiencing different periods of intermittent online teaching, I feel that my capacity to utilize technology in the service of my own teaching has improved.”*	Professional development			
*“At first, some students would leave in the middle of the live broadcast process, then I realized that there would be statistics for online listening, so I sent the listening data to the class group to remind everyone, and then most of the students would finish the complete online course.”*	Platform interactivity	Interactivity	139	32.86%
*“Teachers could not communicate with students face-to-face in online teaching and they could not directly understand the students’ listening status, which led to a lot of information could not be conveyed smoothly in the first time.”*	Teacher-student interaction			
*“Before the online teaching started, we were all worried about how to use Ding Talk, after all, there are many unknowns in online teaching. Luckily, Mr. Wang, the director of the Academic Affairs Office, sent us a timely rain - training teachers on how to use the Ding Talk.”*	Teaching leadership	Instructional leadership and support	24	5.67%
*“In fact, the Education Bureau has provided us with quite a lot of resources, but teachers have to use these resources in their own curriculum, which is suitable for their own students.”*	Resource support			
*“I realize we have to strengthen healthy communication and exchange with parents, I held an online parent-teacher conference, and suggested each family to create an excellent learning environment for their children.”*	Parental support	Home-school collaboration	86	20.33%
*“I communicated promptly with parents on the phone or through WeChat to understand the student’s learning situation, in order to promote students’ learning through home-school cooperation.”*	Parental monitoring			

## 4. Findings

This study explored the effectiveness of online teaching and its influencing factors through semi-structured interviews and the findings are as follows.

### 4.1. Teachers’ acceptance of online teaching

In recent years, the informatization of education has been deepened, and artificial intelligence, big data, cloud computing and other technologies have been integrated and deepened with education. After years of work by information technology in primary and secondary education, it has greatly enriched the teaching methods of schools. During the period of COVID-19, teachers showed their understanding and acceptance of the online teaching, and believed that it was the best way to carry out teaching in special periods.

#### 4.1.1. Perceived usefulness and perceived ease of use of technology influence teachers’ acceptance of online instruction

According to the Technology Acceptance Model (Davis et al., 1989), perceived ease of use and perceived usefulness are decisive factors in the use of a technology system. Perceived usefulness refers to the degree to which the use of a technology system can improve work performance; perceived usability refers to the degree to which the use of a scientific and technological system is convenient and labor-saving. Therefore, from external factors, whether teachers can use relevant software systems to carry out online teaching is largely influenced by perceived ease of use and perceived usefulness. At the beginning of COVID-19, many schools suddenly switched to online teaching, and some teachers had concerns about online teaching, and there were a lot of worries about how to use online teaching software, how to design teaching, presenting teaching content, and how to interact with students, etc. However, with the gradual advancement of online teaching, teachers basically mastered the operation of the online software, and learned to make better use of the online teaching tools to improve their own teaching effectiveness.

#### 4.1.2. Teachers showed their understanding and acceptance to online teaching during special times

During the interviews, it was found that most of the teachers could understand and accepted the use of online teaching during special times. Compared with stopping classes, teachers preferred online teaching to keep students on track and not to make them fall behind. Teachers believed that this was a teaching method that had to be used during special times, but at the same time, it was also the best way to do it. Of course, they also had a lot of concerns about students’ academic performance.

*“As an English teacher and a parent, I think this way of online class is very good, just like my child, she starts to watch online class at eight o’clock every morning, there are two classes in the morning, because online class time is only 20 min a class, it is very easy for children, in the afternoon to do the homework assigned by the teacher, and then take photos and upload, so that children do not fall behind at home. At present, there is no better way to learn. Although there are many problems in this process, it is also the best way to solve the epidemic period of learning.” (Teacher 1)*

### 4.2. Teacher professionalism affects online teaching effectiveness

#### 4.2.1. Teachers’ professional attitudes affect online teaching effectiveness

There is a significant correlation between online teaching efficacy and teachers’ professional attitudes, and online teaching efficacy will be better if teachers are able to carry out online teaching seriously, continue to develop teaching resources, learn the functions of the teaching software, optimize the online teaching design, and adjust the teaching content and teaching methods according to the actual situation. In the interviews, some teachers said that “*teachers are more attentive and detailed in online teaching than in offline classes*.” Of course, this does not represent the practice of all teachers, but in general, the professional attitude of teachers is the most important factor affecting the effectiveness of online teaching.

#### 4.2.2. Teacher professional development impacts online teaching effectiveness

Teachers’ professional development is an important factor affecting the effectiveness of online teaching. In the process of implementing online teaching, some schools provide training for teachers to acquire more online teaching skills, and teachers will learn from each other through peer-to-peer exchanges to improve their online teaching skills. If teachers have the enthusiasm and confidence to continuously improve themselves, their online teaching capacity will be improved more quickly.

*Before the online teaching started, we were all worried about how to use Ding Talk, after all, there are many unknowns in online teaching. Luckily, Mr. Wang, the director of the Academic Affairs Office, sent us a timely rain – training teachers on how to use the Ding Talk. Afterwards, when preparing for the live class, two other teachers in the school and I formed a Ding Talk group to discuss lesson preparation and problems encountered, and through this group, we made several test broadcasts to help each other check. When I initiated the live broadcast for the first time, the class teacher and the language teacher of the second year of junior high school watched the whole lesson online, and found unstable connection and unclear sound to contact me by phone in time to make adjustments. Anyway, in the preliminary stage of taking the live class, many teachers had some problems such as unstable network, lagging, delay, etc. At the end of the live broadcast, we discussed the possible causes of these problems and asked the school’s technical teacher in time. (Teacher 5)*

#### 4.2.3. Teacher professional competence affects online teaching effectiveness

Teachers’ professional competence is a key factor affecting the effectiveness of teachers’ online teaching. Veteran teachers and new teachers have different strengths in online teaching: veteran teachers have more teaching experience, but are not as good as new teachers in the use of online teaching equipment and software, while new teachers are less experienced in teaching, but they are able to make better use of the teaching equipment and software to facilitate their teaching. And both veteran and new teachers face many challenges in online teaching. In the interviews, many teachers mentioned the problems they encountered in online teaching. The professional ability of teachers affects the solution of the problems. How to design and present the teaching content and what teaching methods to use in online teaching can reflect the professional ability of teachers to a large extent.

*The biggest test we faced in online teaching was the adjustment of the teaching content. In the original teaching plan, the teaching module after the midterm exam was interview teaching, we planned to learn the precautions and related requirements before conducting interview activities, and then analyze the setting of interview questions in a certain interview program with the students, and we also designed a simulated interview activity for the students. … Considering many factors, we changed the teaching content and chose to explain some of the knowledge points in General Knowledge of Ancient Chinese Culture. Teachers assign tasks to each student in the class in advance, students explain their own exclusive questions and expand relevant knowledge points, each student has a task to do, and each student shares his or her learning gains, which can ensure the relative fairness of the activity. Students complete the PPT production of their own tasks, unified transmission to the teacher, centralized online display, so that the answers to the study plan and expand the knowledge points more clearly. (Teacher 12)*

During the interviews, it was found that the main teaching methods used by primary and secondary school teachers are relatively diverse, but overall they can be divided into two main categories: live teaching (synchronous) and recorded teaching (asynchronous). In the revised draft, the two teaching methods were compared in terms of lesson time, teacher activities, student activities, teacher-student interactions, and network dependency, as shown in Table 4.

**Table 4:** Comparison of two teaching methods.

Teaching methods	Time	Teachers’ activities	Student activities	Interactivity	Web dependency
Live teaching (synchronous)	Teachers and students online at the same time	Teachers conduct online classes	Students study online at a specified time	Instant Interaction	Vigorous
Recorded teaching (asynchronous)	Teachers and students are not necessarily online at the same time	Teachers play recorded lessons	Students learn course content at any time	Back and forth interaction	Weak

### 4.3. Interactivity: the emotional factor in online teaching effectiveness

Teacher-student interaction is an important aspect of teaching and learning, and teacher-student interaction in online teaching is mainly affected by two factors: the function of the teaching platform; and the communication between teachers and students in terms of cognition, emotion and behavior.

#### 4.3.1. Interactivity support for platform functionality

The interactivity of the teaching platform is the basic condition for teacher-student interaction in online teaching, in order to enhance the presence of online teaching, many teaching platforms have developed a lot of teaching tools, such as live broadcasting is more interactive than recorded broadcasting, and live broadcasting can be checked in, raise your hand, chat, discuss, grab the answer and so on. But sometimes by the stability of the platform, network and other factors, live will appear lag, delay, drop and other conditions. So in online teaching, the teacher on the one hand, to ensure the normal operation of the teacher’s end of the teaching, but also as far as possible to observe the student’s response, these can only be realized through the platform monitoring function and teacher-student interaction.

*Teaching platform is a great impact on teaching, of course, sometimes network reasons, if it is live, there may be a lag, sometimes students themselves will fall off the line, if the fall off the line of his part of the content may not be heard, affecting the back of the course of study, so I recorded the live course, to prevent some students did not hear because of the system or network reasons. … Some software functions are quite good, such as Ding Talk, which is powerful in signing in, monitoring students’ listening to lessons, and correcting homework, etc. There are also cloud classrooms recommended by the Education Bureau, GoodVision, Homework Blackboard, Questionnaire Star, QQ Group, and WeChat Small Programs that can be used to submit homework, which are all available now. There are also some APPs and WeChat small programs that were developed temporarily, which are quite practical, but they may need time to improve. (Teacher 3)*

#### 4.3.2. Online interaction: cognitive, emotional and behavioral communication between teachers and students

When teaching online, different teaching methods will have an impact on teacher-student interactions, and compared with asynchronous classrooms, teachers in synchronous classrooms will have a stronger sense of presence, and will be able to get more timely feedback when conducting online teaching, asking questions, and having discussions. Therefore, in the interviews, some interviewees indicated that they would prefer to use synchronous classroom for teaching, but even so, it was still not observed directly or feel whether the students were able to concentrate on classroom learning and discussion, as they may remain silent during the online course.

*There are a lot of things to break through in online teaching. For example, when I asked a question, some of the younger students could not type and one kept typing periods. Later I realized that many students knew the answer to the question asked by the teacher, but because they could not type, they had no way to express it, and the one who kept typing periods wanted to tell the teacher that he got the question right, and wanted to be praised by the teacher. Therefore, sometimes only a few students interact with the teacher in the online classroom. For those students who type too slowly or cannot type, they may not be able to get encouragement from the online teaching because the teacher does not know their status. (Teacher 4)*

### 4.4. Leading the way: instructional leader support in online teaching and learning

The instructional leadership of educational administration, schools, and academic departments is also one of the important factors affecting online teaching. Especially at the beginning of the implementation of online teaching, when many teachers are full of unknown concerns about online teaching, some schools will utilize their instructional leadership in various ways, such as enhancing teachers’ self-efficacy in online teaching, conducting online teaching training, and providing online teaching resources. Overall, instructional leadership is mainly reflected in two aspects in online teaching: first, the performance of instructional leadership in online teaching; and second, the support of instructional resources in online teaching.

During the interviews, more teachers mentioned the support provided by the educational administration or school for them to conduct online teaching, such as online teaching training and resources, and less mentioned instructional leadership, but these supportive measures for online teaching precisely reflected the instructional leadership of different educational administrators.

*The Department of Education has arranged an online classroom for us, so we can study synchronously, take notes on the lectures in time, draw mind maps, summarize the difficult points and easy-to-mistake knowledge points, and prepare ourselves for online teaching. (Teacher 11)*

*In fact, the Education Bureau has provided us with quite a lot of resources, but teachers have to use these resources in their own curriculum, which is suitable for their own students. (Teacher 3)*

### 4.5. Home-school collaboration: home-school collaboration for students’ learning

What worries many teachers is the non-transparency of the online teaching process and after-school independent learning. When teachers are unable to perceive the presence and reaction of their students in the online classroom and the state of their students in after-school learning, they are filled with worries. Therefore, they need to seek more support from parents to enhance students’ learning through home-school collaboration. During the interviews, a number of teachers mentioned the measures they took to seek parental support to ensure that students’ learning was as effective as possible.

*In the beginning the students in both classes were more conscientious about completing their homework, and then during the second week there was a noticeable slippage. I made an immediate decision to initiate an online meeting with students and parents about the requirements for homework in class, which not only explained my requirements, but also conveyed the idea to parents that parents are their children’s first teachers, and that home-school co-education can only be guaranteed if students’ learning is screwed together to form a united force. Since then, I will be through the WeChat group, Ding Talk group, telephone and other means of timely contact with parents to communicate with their children’s learning situation. (Teacher 5)*

*During the online teaching period, we have held many online parent courses to answer the questions raised by parents, communicate with parents in time about the students’ learning at home, and make reasonable suggestions to parents to provide a fixed and quiet place for their children to study, to really accompany and supervise their children’s learning, to make a learning schedule with their children, and to remind their children of the combination of work and rest and the protection of eye health during the online classes. (Teacher 9)*

## 5. Discussion

This study investigated the factors that influence Chinese primary and secondary school teachers’ online teaching and explored their understanding of these factors. Overall, the majority of primary and secondary school teachers were receptive to online teaching and reported that they were able to understand the significance of online teaching implementation and maintained a positive attitude toward online teaching. Five themes emerged from the interview data: acceptance, professionalism, interactivity, instructional leadership, and home-school collaboration. There were similarities with the results of previous studies (Inusah and Debrah, 2021; Husin et al., 2022).

From the five themes derived from this study, acceptance, professionalism, and interactivity are closely related to individual teachers, while instructional leadership and support, and home-school collaboration are two crucial external support elements.

First, in terms of acceptance of online teaching, this study considers teachers’ acceptance of online teaching as an essential element of the effectiveness of online teaching, and this study shows from the interviews at different times that some teachers were resistant to online teaching when they started to implement online teaching, but with the continuation of online teaching, teachers were able to slowly accept online teaching and show their understanding of online teaching. This has a strong correlation with the use of educational technology, which is consistent with the research of Husin et al. (2022) and Huang et al. (2017). With the improvement of online teaching platforms and teachers’ online teaching ability, teachers will show higher online teaching effectiveness when they are able to carry out online teaching skillfully, and at this time, their acceptance of online teaching will be higher.

Second, the degree of teachers’ professional development affects online teaching effectiveness. Through interviews with teachers at different times of online teaching, this study found that when teachers initially started online teaching, many of them showed different degrees of anxiety, such as the preparation of online teaching materials, and the requirements of parents and schools for students’ performance, etc. However, as teachers developed their online teaching ability, they showed higher acceptance of online teaching. As mentioned by Husin et al. (2022), teachers are prone to stress due to the length of time and deadlines, and if this was already a challenge for them before COVID-19, it became even more so when the classroom shifted to fully online. However, with the development of teachers’ online teaching competencies, teachers have been able to increase their online teaching efficacy by experimenting with different approaches in online teaching, which supports the study of Abdalla Mohammed and Muhammed Pandhiani (2017) that concluded that there is a significant correlation between teachers’ knowledge and teaching competencies and teachers’ teaching efficacy.

Third, teacher-student interaction is an important factor affecting online teaching effectiveness. According to the interview data, teacher-student interaction has always been an important factor troubling teachers’ online teaching. Teachers are worried about online teaching because they are unable to understand the learning status of students in all aspects, and it seems that the learning process of students is a “blind zone.” Although they can see the students’ answers to questions and assignments through technical means, they still cannot see the learning process of the students from various aspects. In order to learn more about the students’ learning dynamics, teachers will increase the number of interactive sessions to capture the students’ attention, and as Garrison et al. (2010) have argued, appropriate socialization activities will support cognitive development.

Fourth, instructional leadership and support provided strong external support for teachers’ online teaching effectiveness. Some of the teachers in the interviews for this study felt that they received great support from educational administration, schools, and peer teachers in the online teaching process, which gave them the confidence and stronger ability to conduct online teaching. This is in line with Husin et al. (2022) who mentioned in their study that the support from the school was more in terms of providing considerable duration and time for teachers’ content, focusing on solving technical problems to simplify the online teaching process, and providing moral support for teachers. It was also evident from the interviews that the school and its departments provided strong technical and moral support for teachers to conduct online teaching.

Fifth, home-school collaboration provided teachers with strong support on the student side for their online teaching effectiveness. Interviews for this study revealed that teachers perceived a greater reliance on support from their families in the online teaching process. If there is a “blind spot” in the interaction between teachers and students in online teaching, then teachers need to obtain information from parents to understand the learning process of students.

## 6. Conclusion

This study deepens the understanding of existing theories of teaching efficacy by identifying and explaining the factors that influence the online teaching effectiveness of Chinese primary and secondary school teachers. The study provides insights for both theory and practice. Previous theories of teaching efficacy do not necessarily may not be applicable in different cultural contexts (Liu et al., 2023). The accountability of these theories may be questioned when they are applied to non-Western developing countries. In addition, their application in educational settings is inadequate (Inusah and Debrah, 2021), especially during COVID-19, and the conduct of online teaching and learning in different countries also presents different characteristics. This study enriches the theory of online teaching efficacy by providing insights into the factors influencing Chinese primary and secondary school teachers’ online teaching.

This study explored the status of Chinese primary and secondary schools conducting online teaching during COVID-19 from both theoretical and empirical perspectives, and extended the theory of teaching efficacy by generalizing new themes from interview data. In terms of practice, this study suggests that the interactive features of teaching platforms should be strengthened to enhance the sense of presence in online teaching; more importantly, teachers should promote online teaching efficacy through continuous professional development. This will help to increase Chinese teachers’ intrinsic motivation to use technology in their future teaching, i.e., to gradually shift from passive obedience to hierarchical power to an intrinsic willingness to integrate technology into teaching. In addition, the interviews expressed the concern that Chinese primary and secondary teachers need to improve their technology, pedagogy, and content knowledge in order to design and use technology well to achieve instructional goals.

## 7. Limitations and future research

This study employed in-depth interviews to explore the factors that influence the online teaching effectiveness of elementary and secondary school teachers, and the sample size was small, which may be unrepresentative of all teachers.

On the other hand, whether the five influential factors derived from this study are significantly related to online teaching efficacy has not been verified, and the five factors can interact with each other, for this reason, the researcher intends to further validate the five derived factors using fsQCA in future studies to explore how different groupings affect online teaching effectiveness.

## Data availability statement

The original contributions presented in the study are included in the article/supplementary material, further inquiries can be directed to the corresponding author.

## Ethics statement

Ethical review and approval was not required for the study on human participants in accordance with the local legislation and institutional requirements. Written informed consent from the participants was not required to participate in this study in accordance with the national legislation and the institutional requirements.

## Author contributions

XM: Conceptualization, Data curation, Formal analysis, Funding acquisition, Investigation, Methodology, Project administration, Resources, Supervision, Validation, Visualization, Writing – original draft, Writing – review & editing.

## Funding

Fujian Provincial Education Science Planning Leading Group Office provides project financial support.Fujian Provincial Education Science “14th Five-Year Plan” 2021 Annual Special Program, Project No.: Fjjgzx21-179.

## Conflict of interest

The author declares that the research was conducted in the absence of any commercial or financial relationships that could be construed as a potential conflict of interest.

## Publisher’s note

All claims expressed in this article are solely those of the authors and do not necessarily represent those of their affiliated organizations, or those of the publisher, the editors and the reviewers. Any product that may be evaluated in this article, or claim that may be made by its manufacturer, is not guaranteed or endorsed by the publisher.
